# A closer look at the high burden of psychiatric disorders among healthcare workers in Egypt during the COVID-19 pandemic

**DOI:** 10.4178/epih.e2021045

**Published:** 2021-07-13

**Authors:** Amr Ehab El-Qushayri, Abdullah Dahy, Abdullah Reda, Mariam Abdelmageed Mahmoud, Sarah Abdel Mageed, Ahmed Mostafa Ahmed Kamel, Sherief Ghozy

**Affiliations:** 1 Faculty of Medicine, Minia University, Minia, Egypt; 2 Faculty of Medicine, Al-Azhar University, Cairo, Egypt; 3 Faculty of Medicine, Tanta University, Tanta, Egypt; 4 Faculty of Pharmacy, Minia University, Minia, Egypt; 5 Faculty of Medicine, Mansoura University, Mansoura, Egypt

**Keywords:** COVID-19, Psychiatry, Mental health, Healthcare workers, Egypt

## Abstract

**OBJECTIVES:**

This study aimed to examine the prevalence of psychiatric disorders among Egyptian healthcare workers (HCWs) during the coronavirus disease 2019 (COVID-19) pandemic.

**METHODS:**

Six databases were searched for relevant papers. The quality of the selected articles was measured using the National Institute of Health quality assessment tool. We used a fixed-effects model when there was no heterogeneity and a random-effects model when there was heterogeneity.

**RESULTS:**

After screening 197 records, 10 studies were ultimately included. Anxiety was the most commonly reported psychiatric disorder among HCWs, with a prevalence of 71.8% (95% confidence interval [CI], 49.4 to 86.9), followed by stress (66.6%; 95% CI, 47.6 to 81.3), depression (65.5%; 95% CI, 46.9 to 80.3), and insomnia (57.9%; 95% CI, 45.9 to 69.0). As measured using the 21-item Depression, Anxiety, and Stress Scale, the most common level of severity was moderate for depression (22.5%; 95% CI, 19.8 to 25.5) and stress (14.5%; 95% CI, 8.8 to 22.9), while high-severity anxiety was more common than other levels of severity (28.2%; 95% CI, 3.8 to 79.6).

**CONCLUSIONS:**

The COVID-19 pandemic has had a negative effect on Egyptian HCWs’ psychological well-being. More psychological support and preventive measures should be implemented to prevent the further development of psychiatric illness among physicians and other HCWs.

## INTRODUCTION

Coronavirus disease 2019 (COVID-19), which emerged in Wuhan, China and is caused by severe acute respiratory syndrome coronavirus 2 (SARS-CoV-2), was declared a global pandemic by the World Health Organization (WHO) in January 2020 [1]. The basic reproductive rate for SARS-CoV-2 was estimated to be around 2.5 (ranging from 1.8 to 3.6), compared with an estimate of 2.0 to 3.0 for SARS-CoV and the influenza pandemic of 1918, 0.9 for Middle East respiratory syndrome coronavirus (MERS-CoV), and 1.5 for the influenza pandemic of 2009 [2]. Thus, SARS-CoV-2 is a highly contagious disease, especially in healthcare settings [1,3]. In Egypt, the number of cases had grown to approximately 158,000 cases and 8,696 deaths as of January 20, 2021 [4]. As a result, quarantine hospitals were designated for COVID-19 cases following a care model recommended and supervised by the WHO. Healthcare workers (HCWs) stay at different quarantine hospitals for consecutive 14-day shifts, followed by polymerase chain reaction (PCR) testing for COVID-19 infection, after which HCWs with negative tests are required to isolate in their homes for another 14 days [4]. HCWs with positive PCR results are admitted to the quarantine hospital at which they worked to receive appropriate medical care [4].

HCWs are the first line of defense during any health crisis, including for past outbreaks of Ebola, scarlet fever, measles, H1N1, H5N1, MERS, and SARS, with the COVID-19 pandemic being the most recent health crisis [5]. During the COVID-19 pandemic, as in previous outbreaks, personal protection is quickly exhausted, workloads are increased, and fears about infection or infection of family members pose a psychological burden on HCWs [6]. Severe anxiety, depression, insomnia, and stress have all been reported by Egyptian HCWs who have treated COVID-19 patients [6,7]. These conditions have a significant effect on HCWs’ work performance, decision-making, and concentration [5], which can lead to work-related diseases and injuries [8]. Moreover, occupational stress has been found to affect HCWs’ intentions to leave work and their professions in the long term [9]. A combination of possible exposure and stress also increases HCWs’ susceptibility to COVID-19 infection [10]. Thus, this study aimed to examine the prevalence of psychiatric disorders among Egyptian HCWs during the COVID-19 outbreak through a systematic review and meta-analysis.

## MATERIALS AND METHODS

### Search strategy and study selection

On December 30, 2020 (updated on January 15, 2021), a systematic search of 6 databases was performed following the PRISMA (Preferred Reporting Items for Systematic Review and Meta-Analyses) guidelines [11]. The 6 databases were Google Scholar, Scopus, the System for Information on Grey Literature in Europe, PubMed, the New York Academy of Medicine, and Web of Science, and the search keywords were (COVID-19 OR COVID 19 OR novel coronavirus) AND (mental health OR depression OR anxiety OR insomnia OR psychology) AND (Egypt). A manual search was conducted after the systematic search to include all relevant papers that may have been missed by the systematic search.

All papers on the prevalence of psychiatric disorders among Egyptian HCWs during the COVID-19 outbreak were included. Studies that examined non-HCWs or non-Egyptians, multicenter studies from which data on Egyptian HCWs could not be extracted, review papers, and conference abstracts were all excluded.

Following the systematic search, duplicate articles were excluded using EndNote version X8 (Thompson Reuters, Carlsbad, CA, USA). Title and abstract screening was initially performed by 2 authors (AEE and AD), and full-text screening was performed by 5 authors (AEE, AR, MAM, SAM, and AMAK). A senior author (SG) was appointed to resolve any conflicts and identify errors.

### Data extraction

A senior author (AEE) developed an extraction template that consisted of 3 parts. The first part was for study characteristics (reference ID, study design, age of participants, sample size, the percentage of male HCWs, the percentage of HCWs who worked in quarantine hospitals, the specific roles of the HCWs, and the scale used for measuring psychiatric disorders). The second part was for the prevalence and severity of psychiatric disorders, and the last part was for the quality rating of each study.

### Risk of bias

All of the included studies were cross-sectional; therefore, the National Institute of Health quality assessment tool was used [12]. We classified the quality of each study into the following 3 categories: 1 for good quality (10-14 points), 2 for fair quality (5-9 points), and 3 for poor quality (0-4 points).

### Statistical analysis

Comprehensive Meta-Analysis version 3 (Biostat Inc., Englewood, NJ, USA) was used to analyze the collected data. We calculated the event rate and its corresponding 95% confidence intervals (CIs) for different psychiatric disorders. Subgroup analysis was conducted based on scores from the 21-item Depression, Anxiety, and Stress Scale (DASS-21), which is the most commonly used scale for measuring psychiatric disorders. Subgroup analysis was not performed when the subgroup consisted of 3 or fewer studies. We used a random-effects model if heterogeneity was found among the pooled results measured using an I^2^ value of > 50% or a p-value of < 0.05 [13]. Otherwise, a fixed-effects model was used. If any outcome was reported in 10 or more studies, a meta-regression or Egger regression (publication bias) analysis was performed [14,15]. Publication bias was considered significant for p-values of < 0.1 in the Egger test.

### Ethics statement

Ethical approval was not applicable as this is a secondary analysis of published papers.

## RESULTS

### Search results and study characteristics

**Figure 1.**
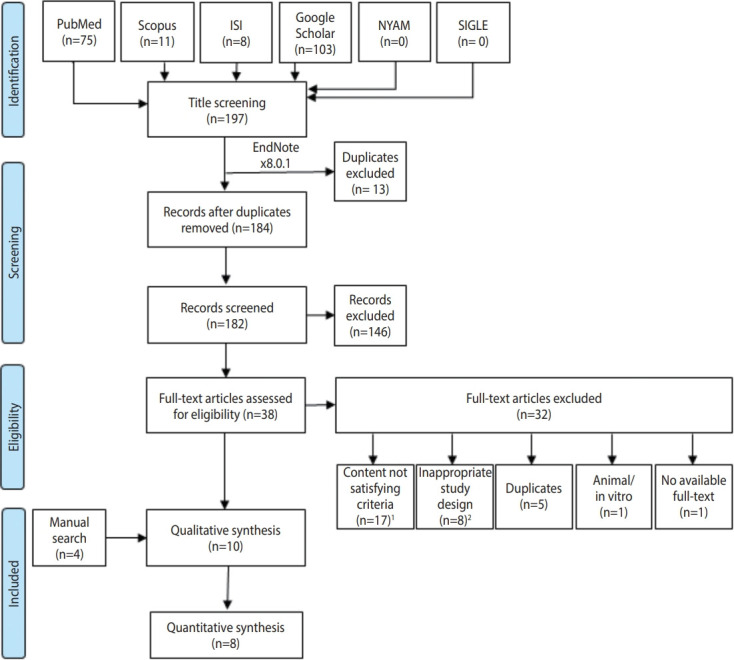
Flow diagram representing the process of the review. ISI, Web of Science; NYAM, New York Academy of Medicine; SIGLE, System for Information on Grey Literature in Europe. ^1^Includes irrelevant articles, no reliable or overlapped data. ^2^Includes conference papers, reviews, books, editorials, letters, oral presentation, correspondence, communications and posters.

In total, 197 records were discovered during the online database search. Of these, 13 were considered duplicates and were excluded before the title and abstract screening. Out of the remaining 184 records, 38 papers were eligible for full-text screening, after which 6 studies were included. Four additional papers were found via a manual search. In total, 10 studies from the database search were included in the analysis (Figure 1 and Table 1) (Supplementary Material 1).

All 10 studies were cross-sectional and had a cumulative sample size of 3,137 HCWs. Six studies included only physicians as participants, 2 included only nurses, one included physicians and nurses, and one included physicians, nurses, pharmacists, house officers, technicians, and others (Table 1).

### Anxiety

**Figure 2.**
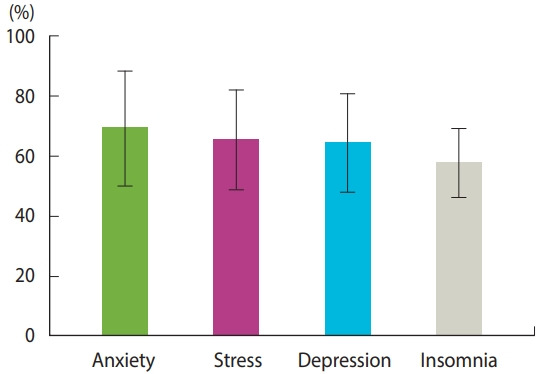
The prevalence of psychiatric disorders represented by the event rate and corresponding 95% confidence interval.

**Figure 3.**
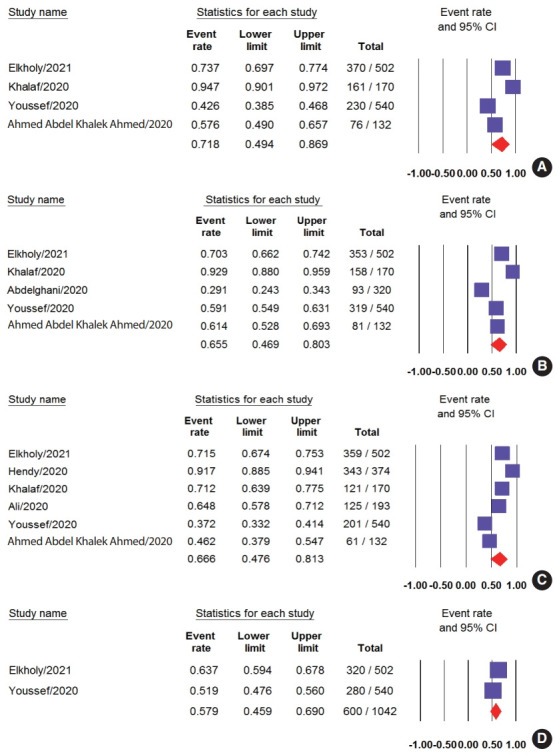
The prevalence of (A) total anxiety, (B) total depression, (C) total stress, and (D) total insomnia represented by the event rate and the corresponding 95% confidence interval (CI).

Of the 4 studies that assessed anxiety among HCWs, 3 used the DASS-21 [4,16,17], and one used the Patient Health Questionnaire (PHQ) [6]. The overall prevalence of anxiety was high, at 71.8% (95% CI, 49.4 to 86.9), with 28.2% (95% CI, 3.8 to 79.6) of the participants reporting severe forms of anxiety (Figures 2 and 3A). Moderate, mild, and severe anxiety had prevalence rates of 12.3% (95% CI, 6.1 to 23.1), 9.9% (95% CI, 4.9 to 19.0), and 6.4% (95% CI, 4.9 to 8.3), respectively (Supplementarty Materials 2-5). A random-effects model was adopted for all analyses due to significant heterogeneity (p < 0.001) except for severe anxiety, for which there was no heterogeneity (p= 0.42).

### Depression

Five studies examined the prevalence of depression among HCWs, with 3 of them using the DASS-21 scale [4,16,17], 1 using the PHQ [6], and 1 using the Hospital Anxiety and Depression Scale [18]. The pooled depression prevalence was 65.5% (95% CI, 46.9 to 80.3); however, the results showed significant heterogeneity (p< 0.001, I^2^ = 98%) (Figures 2 and 3B). Moderate depression was the most common type of depression in terms of severity, with a prevalence of 22.5% (95% CI, 19.8 to 25.5), followed by very severe depression (16.3%; 95% CI, 5.4 to 39.9), severe depression (16.1%; 95% CI, 8.3 to 28.9), and mild depression (12.5%; 95% CI, 8.0 to 19.0) (Supplementary Materials 6-9). Nevertheless, significant heterogeneity was found for severe and mild depression (p< 0.001), while moderate depression showed no heterogeneity (p= 0.42).

### Stress

Seven studies examined stress experienced by HCWs. Three studies used the DASS-21 [4,16,17], 2 studies used the Perceived Stress Scale [6,19], and 2 used the Nursing Stress Scale [7,20]. The pooled prevalence of stress was 66.6% (95% CI, 47.6 to 81.3) (Figures 2 and 3C). Moderate stress was the most common type of stress experienced by HCWs (14.5%; 95% CI, 8.8 to 22.9) followed by severe, mild, and very severe stress, with rates of 14.3% (95% CI, 10.0 to 20.1), 11.5% (95% CI, 9.5 to 13.9), and 9.3% (95% CI, 3.2 to 24.0), respectively (Supplementary Materials 10-13). We used a random-effects model to analyze the total prevalence and severity of anxiety due to the presence of significant heterogeneity (p< 0.001) except for moderate severity, for which significant heterogeneity was not found.

### Insomnia

Insomnia was examined in 2 studies, both of which measured insomnia using the Insomnia Severity Index [4,6]. The rate of insomnia among HCWs was 57.9% (95% CI, 45.9 to 69.0) (Figures 2 and 3D).

### Burnout

One study measured the prevalence of burnout among 220 physicians using the Maslach Burnout Inventory Human Services Survey [21]. The prevalence of burnout was 36%.

### Distress

One study examined the prevalence of distress among 266 HCWs, which was measured using the Peritraumatic Distress Index [22]. The total prevalence of distress was 67%. Of those with distress, 52% experienced mild and moderate distress and 15% experienced severe distress.

### Death ideation

Only one study examined death ideation among HCWs. Across a total of 320 physicians, 35 physicians reported having death ideation, with a prevalence of 11% [18].

## DISCUSSION

Under normal circumstances, the risk of HCWs developing a psychiatric disorder is higher than that of workers in other occupations and the general population [23,24]. During the COVID-19 pandemic, the risk is even higher as HCWs are required to make major psychological and physical efforts to face the burden caused by the pandemic. Such burdens can have a major impact on the quality of care provided to patients due to an increase in medical errors, which in turn further increase the burden on healthcare systems [25-27].

In the present study, we aimed to gather further evidence on the prevalence of psychiatric disorders among Egyptian HCWs to draw attention to their experiences, provide support to address their struggles, and improve the quality of provided care. Our analysis showed that the prevalence of reported psychological symptoms was notably high, as previously mentioned. The rates of psychological illness found in this study were much higher than those found in studies about HCWs in other countries, including China [28] and Italy [29]. In Singapore and India, a similar multinational study was conducted and found that the prevalence rates for anxiety, depression, and stress were 15.7%, 10.6%, and 5.2%, respectively, which are much lower than the estimated prevalence rates found in the present study [30]. Another meta-analysis found that the prevalence rates of anxiety and depression were 23.2% and 22.8%, respectively [31]. A more recent meta-analysis also found similar prevalence rates of psychiatric illnesses among HCWs, with estimated prevalence rates of 24.3%, 25.8%, and 45.0% for depression, anxiety, and stress, respectively [32]. Comparing these numbers to our results clearly shows the degree to which the Egyptian healthcare system was burdened by the COVID-19 pandemic and indicates an urgent need for effective solutions to reduce the burden.

The prevalence of psychiatric symptoms was higher among HCWs in Egypt during the COVID-19 pandemic than in the preCOVID-19 era. A cross-sectional study conducted at Alexandria University that recruited nurses in critical care units found prevalence rates of 51%, 69%, and 50% for depression, anxiety, and stress, respectively, among nurses [33]. In addition, Al-Sayed et al. [34] conducted a cross-sectional study on residents at Ain Shams University that found that 53% of residents were affected with mild to moderate depression. After the start of the COVID-19 pandemic, and according to our results, there has been a noticeable rise in psychiatric symptoms experienced by HCWs, including anxiety (71.8%), stress (66.6%), depression (65.5%), and insomnia (57.9%).

Many reasons can be attributed to the high estimated prevalence of psychological disorders among HCWs. First, the effects of the pandemic and the negative emotions related to the millions of people who were infected and subsequent hundreds of thousands of deaths. Such feelings are heightened for HCWs, who are in direct contact with COVID-19 patients [10,35]. Such feelings are possibly even worse among HCWs who work in areas other than infectious diseases since they may lack experience and not know how to deal appropriately with respiratory illnesses or use personal protective equipment [36]. In addition, Egyptian HCWs are subject to intensive workloads, including potential 24-hour shifts, to compensate for the shortage of HCWs in the Egyptian healthcare system. This, combined with shortages in medical supplies and low wages received by Egyptian HCWs, are potential risk factors for developing psychological disorders. Moral distress is another possible explanation for the decrease in the mental health of HCWs since they are forced to work only with the resources available to them [35,37]. Lack of direct support for HCWs from supervisors and a heightened culture of blame for potential mistakes is also a potential factor that influences the prevalence of psychiatric disorders among HCWs [7]. Additionally, HCWs may be exposed to unexplained stress and even violence from patients and their families due to the decreased quality of care [8]. Social stigmatization is also a factor that influences HCWs’ mental health since people may have safety concerns about interacting with HCWs who are in direct contact with COVID-19 patients [38].

Our findings indicate an urgent need for proper interventions to increase the quality of care in Egyptian healthcare facilities. The Egyptian Ministry of Health already provides a direct hotline for HCWs to report their needs and make urgent requests. Further measures should also be urgently undertaken to decrease the high prevalence rate of psychological disorders among HCWs. We suggest that HCWs should be vaccinated as efficiently as possible and receive adequate personal protective equipment to reduce their risk of COVID-19 infection [39]. Improving the quality of life of HCWs might also relieve HCWs’ psychological symptoms and encourage them to keep working in Egypt’s healthcare system [40-42]. Organizing the daily shifts of HCWs and better distributing their work hours are also recommended; however, this should be done based on available resources [43].

Our results are limited to the small number of studies included in our analysis and the small sample size of the included studies. Moreover, heterogeneity in our results may be a result of differences in proportion of male HCWs, the role of HCWs, and the scales used to measure psychiatric illnesses in the included studies.

## CONCLUSION

The COVID-19 pandemic has had an unfavorable effect on the psychological well-being of Egyptian HCWs. More psychological support and preventive measure should be implemented to prevent HCWs from developing major psychiatric illnesses. Collaborative efforts between policymakers, hospital executives, members of the media, and the general population are urgently needed to address this issue.

## Figures and Tables

**Table 1.** Characteristics of the included studies

Study	Study design	Works in quarantine hospital, n (%)	Sample size	Age (mean ±SD or range), yr	Sex, male	Type of health care workers (%)	Risk of bias
Elkholy et al., 2021 [6]	Cross-sectional	237 (47.2)	502	>18	251	Physicians (60.0), nurses (40.0)	Fair
Abdelhafiz et al., 2020 [21]	Cross-sectional	NR	220	33.42±5.3	111	Physicians (100)	Fair
Hendy et al., 2020 [20]	Cross-sectional	374 (100)	374	32.06±3.9	122	Nurses (100)	Fair
Khalaf et al., 2020 [16]	Cross-sectional	NR	170	36.47±5.1	66	Physicians (100)	Fair
Abdelghani et al., 2020 [18]	Cross-sectional	NR	320	34.6±6.0	117	Physicians (100)	Fair
Said et al., 2021 [7]	Cross-sectional	210 (50.0)	420	20-59	76	Nurses (100)	Fair
Ali et al., 2020 [19]	Cross-sectional	NR	193	NR	NR	Physicians (100)	Fair
Youssef et al., 2020 [4]	Cross-sectional	55 (10.2)	540	37.3±9.2	294	Physicians (77.0), nurses (9.0), pharmacists (7.0), house officer (2.0), Technician (2.0), other (3.0)	Fair
Abasiri et al., 2020 [22]	Cross-sectional	NR	266	NR	NR	NR	Fair
Ahmed Abdel Khalek Ahmed et al., 2020 [17]	Cross-sectional	NR	132	24.6	3.3	Nurses (100)	Fair

SD, standard deviation; NR, not reported.

## SUPPLEMENTARY MATERIALS

Supplementary Material 1. Quality rating of the prospective cohort studies

Supplementary Material 2. The prevalence of mild anxiety represented by the event rate and the corresponding 95% confidence interval (CI)

Supplementary Material 3. The prevalence of moderate anxiety represented by the event rate and the corresponding 95% confidence interval (CI)

Supplementary Material 4. The prevalence of severe anxiety represented by the event rate and the corresponding 95% confidence interval (CI)

Supplementary Material 5. The prevalence of very severe anxiety represented by the event rate and the corresponding 95% confidence interval (CI)

Supplementary Material 6. The prevalence of mild depression represented by the event rate and the corresponding 95% confidence interval (CI)

Supplementary Material 7. The prevalence of moderate depression represented by the event rate and the corresponding 95% confidence interval (CI)

Supplementary Material 8. The prevalence of severe depression represented by the event rate and the corresponding 95% confidence interval (CI)

Supplementary Material 9. The prevalence of very severe depression represented by the event rate and the corresponding 95% confidence interval (CI)

Supplementary Material 10. The prevalence of mild stress represented by the event rate and the corresponding 95% confidence interval (CI)

Supplementary Material 11. The prevalence of moderate stress represented by the event rate and the corresponding 95% confidence interval (CI)

Supplementary Material 12. The prevalence of severe stress represented by the event rate and the corresponding 95% confidence interval (CI)

Supplementary Material 13. The prevalence of very severe stress represented by the event rate and the corresponding 95% confidence interval (CI)
