# Supplementary material for: A closer look at the high burden of psychiatric disorders among healthcare workers in Egypt during the COVID-19 pandemic
Source: Epidemiol Health. 2021 Jul 13;43:e2021045. doi: 10.4178/epih.e2021045 (PMC8602011; doi:10.4178/epih.e2021045)
Supplement: Supplementary Material 1. — Quality rating of the prospective cohort studies [file epih-43-e2021045-suppl1.pdf]

Volume: 43, Article ID: e2021045

https://doi.org/10.4178/epih.e2021045

Supplementary Material 1. Quality rating of the prospective cohort studies

Reference ID	Q1	Q2	Q3	Q4	Q5	Q6	Q7	Q8	Q9	Q10	Q11	Q12	Q13	Q14	Total score
Elkholy/2020	1	1	0	1	0	1	1	0	1	1	1	0	0	1	9
Abdelhafiz/2020	1	1	1	1	0	1	0	1	1	0	1	0	0	1	9
Hendy/2020	1	1	1	1	0	1	0	1	1	0	1	0	0	1	9
Khalaf/2020	1	1	1	1	0	1	0	1	1	0	1	0	0	1	9
Abdelghani/2020	1	1	1	1	1	1	0	1	1	0	1	0	0	1	10
Said/2020	1	1	1	1	1	1	0	1	1	0	1	0	0	1	10
Ali/2020	1	1	1	1	1	1	0	1	1	0	1	0	0	1	10
Youssef/2020	1	1	1	1	0	1	0	1	1	0	1	0	0	1	9
El-Abasiri/2020	1	1	1	1	1	1	0	1	1	0	1	0	0	1	10
Ahmed/2021	1	1	1	1	0	1	0	1	1	0	1	0	0	1	9

1 = yes, 0 = no, not applicable or not reported
Goo quality = 11-14 points, fair quality = 6-10 points and poor quality = 0-5 points.

1- Was the research question or objective in this paper clearly stated?
2- Was the study population clearly specified and defined?
3- Was the participation rate of eligible persons at least 50%?
4- Were all the subjects selected or recruited from the same or similar populations (including the same time period)? Were inclusion and exclusion criteria for being in the study prespecified and applied uniformly to all participants?
5- Was a sample size justification, power description, or variance and effect estimates provided?
6- For the analyses in this paper, were the exposure(s) of interest measured prior to the outcome(s) being measured?
7- Was the timeframe sufficient so that one could reasonably expect to see an association between exposure and outcome if it existed?
8- For exposures that can vary in amount or level, did the study examine different levels of the exposure as related to the outcome (e.g., categories of exposure, or exposure measured as continuous variable)?
9- Were the exposure measures (independent variables) clearly defined, valid, reliable, and implemented consistently across all study participants?
10- Was the exposure(s) assessed more than once over time?
11- Were the outcome measures (dependent variables) clearly defined, valid, reliable, and implemented consistently across all study participants?
12- Were the outcome assessors blinded to the exposure status of participants?
13- Was loss to follow-up after baseline 20% or less?
14- Were key potential confounding variables measured and adjusted statistically for their impact on the relationship between exposure(s) and outcome(s)?
