# Supplementary material for: A closer look at the high burden of psychiatric disorders among healthcare workers in Egypt during the COVID-19 pandemic
Source: Epidemiol Health. 2021 Jul 13;43:e2021045. doi: 10.4178/epih.e2021045 (PMC8602011; doi:10.4178/epih.e2021045)

Volume: 43, Article ID: e2021045

https://doi.org/10.4178/epih.e2021045

Supplementary Material 2. The prevalence of mild anxiety represented by the event rate and the corresponding 95% confidence interval (CI)

Study name	Statistics for each study				Event rate and 95% CI
	**Event rate**	**Lower limit**	**Upper limit**	**Total**	
Khalaf/2020	0.041	0.020	0.084	7 / 170	
Youssef/2020	0.161	0.132	0.195	87 / 540	
Ahmed/2021	0.114	0.070	0.180	15 / 132	
	0.099	0.049	0.190		

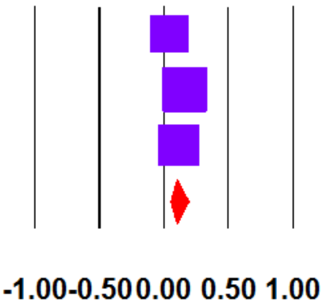

Supplementary Material 3. The prevalence of moderate anxiety represented by the event rate and the corresponding 95% confidence interval (CI)

Study name	Statistics for each study				Event rate and 95% CI
	**Event rate**	**Lower limit**	**Upper limit**	**Total**	
Khalaf/2020	0.059	0.032	0.106	10 / 170	
Youssef/2020	0.115	0.091	0.145	62 / 540	
Ahmed/2021	0.235	0.170	0.315	31 / 132	
	0.123	0.061	0.231		

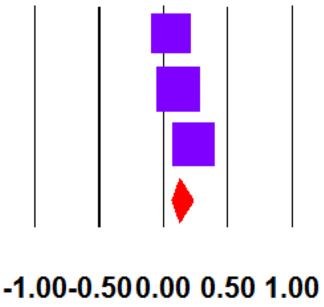

Supplement: Supplementary Material 2. — The prevalence of mild anxiety represented by the event rate and the corresponding 95% confidence interval (CI) [file epih-43-e2021045-suppl2.pdf]
