# Supplementary material for: A closer look at the high burden of psychiatric disorders among healthcare workers in Egypt during the COVID-19 pandemic
Source: Epidemiol Health. 2021 Jul 13;43:e2021045. doi: 10.4178/epih.e2021045 (PMC8602011; doi:10.4178/epih.e2021045)

Volume: 43, Article ID: e2021045

https://doi.org/10.4178/epih.e2021045

Supplementary Material 4. The prevalence of severe anxiety represented by the event rate and the corresponding 95% confidence interval (CI)

Study name	Statistics for each study			
	**Event rate**	**Lower limit**	**Upper limit**	**Total**
Khalaf/2020	0.071	0.041	0.120	12 / 170
Youssef/2020	0.056	0.039	0.078	30 / 540
Ahmed/2021	0.083	0.047	0.144	11 / 132
	0.064	0.049	0.083	

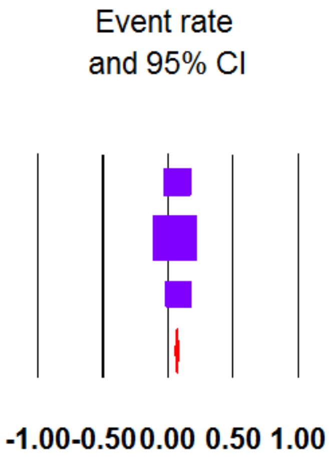

Supplementary Material 5. The prevalence of very severe anxiety represented by the event rate and the corresponding 95% confidence interval (CI)

Study name	Statistics for each study			
	**Event rate**	**Lower limit**	**Upper limit**	**Total**
Khalaf/2020	0.776	0.708	0.833	132 / 170
Youssef/2020	0.094	0.072	0.122	51 / 540
Ahmed/2021	0.144	0.094	0.215	19 / 132
	0.282	0.038	0.796	

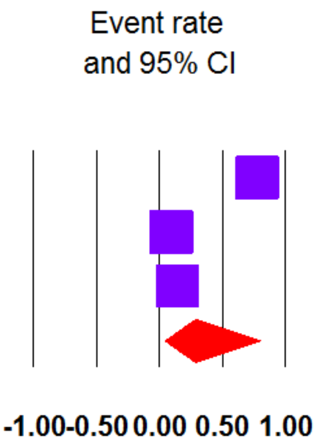

Supplement: Supplementary Material 4. — The prevalence of severe anxiety represented by the event rate and the corresponding 95% confidence interval (CI) [file epih-43-e2021045-suppl3.pdf]
