# Supplementary material for: A closer look at the high burden of psychiatric disorders among healthcare workers in Egypt during the COVID-19 pandemic
Source: Epidemiol Health. 2021 Jul 13;43:e2021045. doi: 10.4178/epih.e2021045 (PMC8602011; doi:10.4178/epih.e2021045)

Volume: 43, Article ID: e2021045

https://doi.org/10.4178/epih.e2021045

Supplementary Material 6. The prevalence of mild depression represented by the event rate and the corresponding 95% confidence interval (CI)

Study name	Statistics for each study			
	Event rate	Lower limit	Upper limit	Total
Khalaf/2020	0.106	0.068	0.162	18 / 170
Youssef/2020	0.172	0.143	0.206	93 / 540
Ahmed/2021	0.091	0.052	0.153	12 / 132
	0.125	0.080	0.190	

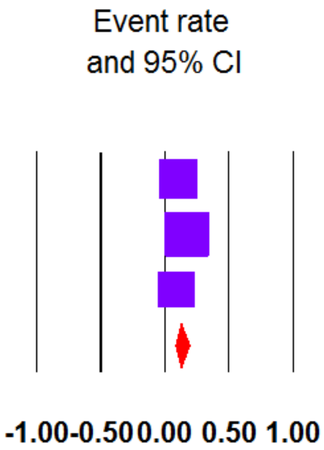

Supplementary Material 7. The prevalence of moderate depression represented by the event rate and the corresponding 95% confidence interval (CI)

Study name	Statistics for each study			
	Event rate	Lower limit	Upper limit	Total
Khalaf/2020	0.194	0.141	0.260	33 / 170
Youssef/2020	0.226	0.193	0.263	122 / 540
Ahmed/2021	0.258	0.190	0.339	34 / 132
	0.225	0.198	0.255	

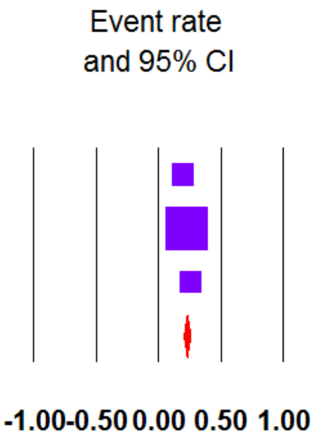

Supplement: Supplementary Material 6. — The prevalence of mild depression represented by the event rate and the corresponding 95% confidence interval (CI) [file epih-43-e2021045-suppl4.pdf]
