# Supplementary material for: A closer look at the high burden of psychiatric disorders among healthcare workers in Egypt during the COVID-19 pandemic
Source: Epidemiol Health. 2021 Jul 13;43:e2021045. doi: 10.4178/epih.e2021045 (PMC8602011; doi:10.4178/epih.e2021045)

Volume: 43, Article ID: e2021045

https://doi.org/10.4178/epih.e2021045

Supplementary Material 8. The prevalence of severe depression represented by the event rate and the corresponding 95% confidence interval (CI)

Study name	Statistics for each study			
	Event rate	Lower limit	Upper limit	Total
Khalaf/2020	0.259	0.199	0.330	44 / 170
Youssef/2020	0.098	0.076	0.126	53 / 540
Ahmed/2021	0.159	0.106	0.232	21 / 132
	0.161	0.083	0.289	

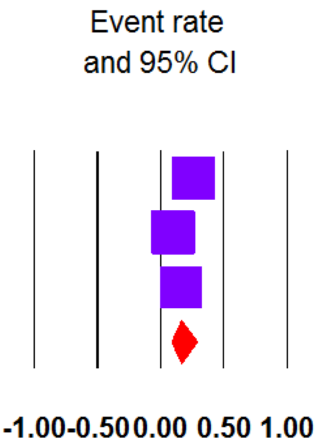

Supplementary Material 9. The prevalence of very severe depression represented by the event rate and the corresponding 95% confidence interval (CI)

Study name	Statistics for each study			
	Event rate	Lower limit	Upper limit	Total
Khalaf/2020	0.371	0.301	0.446	63 / 170
Youssef/2020	0.094	0.072	0.122	51 / 540
Ahmed/2021	0.106	0.064	0.171	14 / 132
	0.163	0.054	0.399	

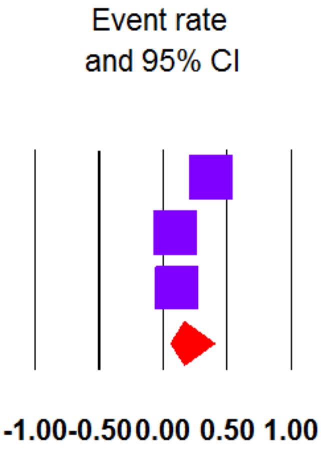

Supplement: Supplementary Material 8. — The prevalence of severe depression represented by the event rate and the corresponding 95% confidence interval (CI) [file epih-43-e2021045-suppl5.pdf]
