# Supplementary material for: A closer look at the high burden of psychiatric disorders among healthcare workers in Egypt during the COVID-19 pandemic
Source: Epidemiol Health. 2021 Jul 13;43:e2021045. doi: 10.4178/epih.e2021045 (PMC8602011; doi:10.4178/epih.e2021045)

Volume: 43, Article ID: e2021045

https://doi.org/10.4178/epih.e2021045

Supplementary Material 10. The prevalence of mild stress represented by the event rate and the corresponding 95% confidence interval (CI)

Study name	Statistics for each study			
	Event rate	Lower limit	Upper limit	Total
Khalaf/2020	0.118	0.077	0.175	20 / 170
Youssef/2020	0.122	0.097	0.153	66 / 540
Ahmed/2021	0.076	0.041	0.135	10 / 132
	0.115	0.095	0.139	

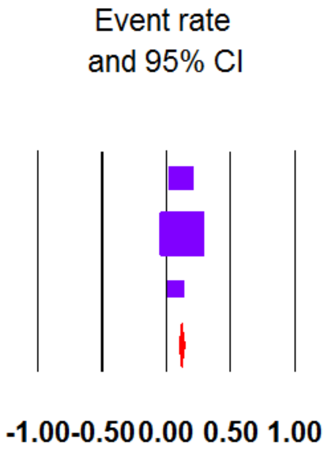

Supplementary Material 11. The prevalence of moderate stress represented by the event rate and the corresponding 95% confidence interval (CI)

Study name	Statistics for each study			
	Event rate	Lower limit	Upper limit	Total
Khalaf/2020	0.206	0.152	0.273	35 / 170
Youssef/2020	0.098	0.076	0.126	53 / 540
Ahmed/2021	0.152	0.100	0.223	20 / 132
	0.145	0.088	0.229	

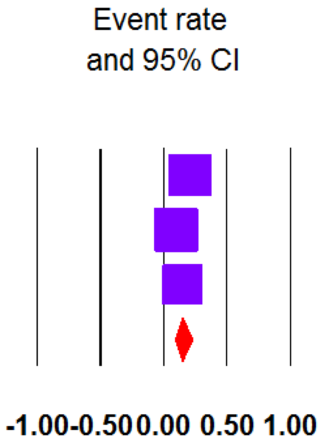

Supplement: Supplementary Material 10. — The prevalence of mild stress represented by the event rate and the corresponding 95% confidence interval (CI) [file epih-43-e2021045-suppl6.pdf]
