# Supplementary material for: A closer look at the high burden of psychiatric disorders among healthcare workers in Egypt during the COVID-19 pandemic
Source: Epidemiol Health. 2021 Jul 13;43:e2021045. doi: 10.4178/epih.e2021045 (PMC8602011; doi:10.4178/epih.e2021045)

Volume: 43, Article ID: e2021045

https://doi.org/10.4178/epih.e2021045

Supplementary Material 12. The prevalence of severe stress represented by the event rate and the corresponding 95% confidence interval (CI)

Study name	Statistics for each study			
	**Event rate**	**Lower limit**	**Upper limit**	**Total**
Khalaf/2020	0.194	0.141	0.260	33 / 170
Youssef/2020	0.113	0.089	0.143	61 / 540
Ahmed/2021	0.136	0.088	0.206	18 / 132
	0.143	0.100	0.201	

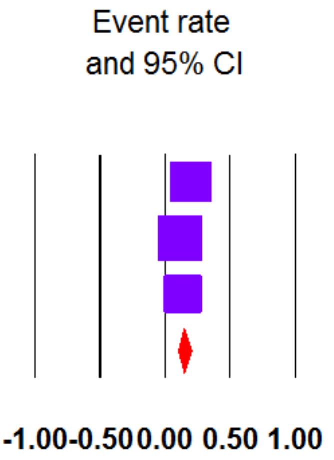

Supplementary Material 13. The prevalence of very severe stress represented by the event rate and the corresponding 95% confidence interval (CI)

Study name	Statistics for each study			
	**Event rate**	**Lower limit**	**Upper limit**	**Total**
Khalaf/2020	0.194	0.141	0.260	33 / 170
Youssef/2020	0.039	0.025	0.059	21 / 540
Ahmed/2021	0.098	0.058	0.162	13 / 132
	0.093	0.032	0.240	

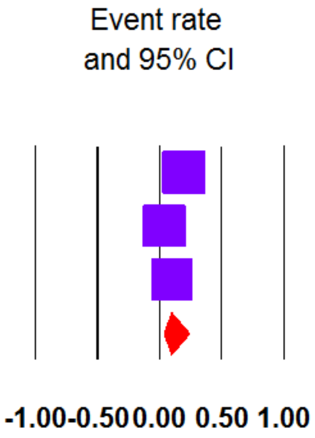

Supplement: Supplementary Material 12. — The prevalence of severe stress represented by the event rate and the corresponding 95% confidence interval (CI) [file epih-43-e2021045-suppl7.pdf]
